# EHMTI-0167. Prevalence of migraine in schoolchildren in the republic of Moldova. Pilot study

**DOI:** 10.1186/1129-2377-15-S1-B29

**Published:** 2014-09-18

**Authors:** T Lozan, S Odobescu, L Rotaru, I Moldovanu, O Grosu

**Affiliations:** 1 Neurology, Institute of Neurology and Neurosurgery, Chisinau, Moldova; 2 Neurology, State Medical and Pharmaceutical University "N.Testemitanu", Chisinau, Moldova

## Background

Migraine is a disabling primary headache that often has its onset in childhood and adolescence and may be present in 7-10 % of children by age 15.

## Aim

To study the prevalence of migraine in adolescents in the Republic of Moldova (RM).

## Methods

The 6-month period study included 500 schoolchildren aged between 14 –19 years from randomly choosed schools in Chishinau, capital city of the RM. We developed a special questionnaire including 10 chapters (101 questions). The headache diagnosis was established based on the ICHD-II (2004) and ICHD-IIR (2006) criteria. The data were analysed using SPSS software.

## Results

Of 500 schoolchildren interviewed 338 (67.6%) were suffering from headache and 162 (32.4%) were headache free. The average age of respondents was 16.4±0.17 years. The average age of headache onset was 12.1±0.23 years. Migraine distribution by gender was as follows: girls - 73.4% and boys – 26.6%. The prevalence of migraine was 19.4%, with episodic migraine in 11.6% and chronic migraine – in 7.8% cases. Regarding age criterion the questionnaires were distributed as follows: 14 years – 16 (3.2%) persons, 15 years – 101 (20.2%) persons, 16 years – 151 (30.2%) persons, 17 years – 117 (23.4%) persons, 18 years – 108 (21.6%) persons, 19 years – 7 (1.4%) persons. According to gender, there were 314 (62.8%) girls and 186 (37.2%) boys.

## Conclusion

The preliminary data show a quite high prevalence of episodic (11.6%) and chronic (7.8%) migraine among schoolchildren of the Republic of Moldova. Further analysis with bigger sample size will offer more accuracy.

No conflict of interest.

